# Use of headphones for the delivery of music programs for people with dementia in long-term care homes: a scoping review

**DOI:** 10.3389/frdem.2025.1707201

**Published:** 2026-01-16

**Authors:** Yaqi Huang, Karen Lok Yi Wong, Daphne Sze Ki Cheung, Myung Sun Yeo, Soo Ji Kim, Macdonald Sue, Lillian Hung

**Affiliations:** 1 School of Nursing, The Hong Kong Polytechnic University, Kowloon, Hong Kong SAR, China; 2 IDEA Lab, The University of British Columbia, Vancouver, BC, Canada; 3 Centre for Quality and Patient Safety Research, Alfred Health Partnership, Institute for Health Transformation, Geelong, VIC, Australia; 4 School of Nursing and Midwifery, Deakin University, Melbourne, VIC, Australia; 5 Music Therapy Education, Ewha Womans University, Seoul, Republic of Korea; 6 Vancouver Coastal Health Authority, Vancouver, BC, Canada

**Keywords:** headphones, music therapy, dementia, long-term care, barriers, facilitators

## Abstract

**Objective:**

To examine the evidence regarding to the use of headphones in music programs for people with dementia in long-term care homes (LTC) and identify enablers and barriers to its implementation.

**Introduction:**

Headphones can provide an immersive auditory experience, powerfully stimulating memories and evoking emotional expression. However, reviews on factors influencing their implementation in LTC settings are limited.

**Methods:**

This review followed the Joanna Briggs Institute methodology and was reported according to the PRISMA-ScR Checklist. A search was conducted across databases, including PubMed, CINAHL, Embase, Web of Science, Scopus, PsycINFO, and ProQuest. Studies were included if they explicitly delivered the music program using headphones for people with dementia in LTC homes. Data were extracted and thematically synthesized to identify key enablers and barriers to headphone use and program implementation.

**Results:**

A total of 21 studies were included. Music delivered via headphones demonstrated significant potential to pain relief, reduce the behavioral and psychological symptoms, delirium, control hyperactive behavior, and improve sleep quality. The key enablers for its implementation included (1) Comfortable and immersive experience, (2) Good accessibility and sustainability, (3) Enhanced engagement and interactions and (4) Appropriate staff training and collaboration. Barriers included (a) Less optimal headphone options in dementia care, (b) Staff burden and shortage, (c) Operational challenges, and (d) Music selection and personalization challenges.

**Conclusion:**

This scoping review identifies key enablers and barriers to implementing headphone-based music programs for people with dementia in LTC homes. Future research should develop strategies for optimal headphone use, staff collaboration, and personalized music delivery to support sustainable and effective implementation.

## Introduction

Music has been recognized in the literature as a non-pharmacological intervention to support the cognitive and psychosocial well-being of older adults living with dementia (Paraskevopoulos, 2023). Research has demonstrated that music intervention can alleviate chronic pain (Tse et al., 2023), reduce negative behaviors (Pedersen et al., 2017), improve mood (Paraskevopoulos, 2023), alleviate symptoms of anxiety, depression (Cheung et al., 2020, 2022), and agitation of people with dementia (Cheung et al., 2023; Pedersen et al., 2017). Remarkably, the review by Garrido et al. (2017) demonstrated that pre-recorded music can be effective in reducing agitation without a trained music therapist.

Long-term care (LTC) homes, including nursing homes and hospitals, can be noisy environments with noises from both human and non-human sources for older adults living with dementia. A systematic review by Janus et al. (2021) showed that the noises in LTC homes can negatively affect the quality of life of residents living with dementia, such as reducing their nighttime sleep and increasing their agitation. One approach to mitigating these disturbances is the use of headphones for listening to music, which can create a more focused and personalized listening experience than using loudspeakers (Locke and Mudford, 2010). In the study in an older adult mental health unit by Hung et al. (2021), listening to music of patients’ choices with quality audio via silent disco headphones was found to reduce distractions to patients and help them maintain focus. Particularly for people with dementia, the immersive music experience created by headphones can bring moments of joy, even in the face of cognitive decline (Corrêa et al., 2020). Moreover, when the immersive experience is delivered in a group setting, it can encourage emotional expression and foster social connections (Harrison et al., 2021; Hung et al., 2021).

Although delivering music using headphones shows promise in improving the well-being of residents in care settings, Li et al. (2015) reported a high drop-out rate of 61.5% of older adults in a program listening to music using headphones. Gaviola et al. (2022) explored the challenges of implementing headphones for music intervention and found that older adults and service providers hesitated to use headphones because older adults felt uncomfortable wearing them, and service providers had challenges accessing them.

While recent advancement in headphone design, such as lighter materials and wireless connectivity have improved headphones usability in LTC settings for people with dementia (Kwak et al., 2021; Weise et al., 2019), the adoption remians inconsistent. Programs like Music and Memory (M&M), which implemented over-the-ear headphones or earphones among residents with dementia in Wisconsin nursing homes, have demonstrated behavioral improvements in some studies (Kwak et al., 2016, 2021), yet other studies reported limited or non-significant effect on agitation and mood (Kwak et al., 2016; Locke and Mudford, 2010). Additionally, LTC staff also expressed concerns about the sustainability and operational burden (Kwak et al., 2016).

While prior research has examined the general effectiveness of headphone-based music programs for people with dementia, there remains a limited understanding of the enablers and barriers to their implementation in LTC settings. Additionally, existing studies rarely provide concrete recommendations on how to utilize enablers to address barriers related to headphone selection and management, staff training, and music delivery. These gaps hinder the real-world applicability of headphone-based programs. Therefore, this scoping review aims to explore the use of headphones in music programs for people with dementia in LTC homes, with a focus on identifying enablers and barriers to their implementation. The findings will provide practical insights for staff, researchers, caregivers, policymakers, offering strategies to enhance the adoption, acceptability and sustainability of headphone-based music interventions in dementia care.

## Materials and methods

The scoping review follows the Joanna Briggs Institute (JBI) methodology for scoping reviews (Peters et al., 2020). The review was conducted in accordance with the protocol we published previously (Hung et al., 2024) and the reporting followed the Preferred Reporting Items for Systematic Reviews and Meta-analysis Protocols Extension for Scoping Reviews (PRISMA-ScR) (Tricco et al., 2018), which is present in the Supplementary File 1. Studies from January 2010 to March 2024 were considered for inclusion based on the selection criteria listed below (see Table 1).

**Table 1:** Inclusion and exclusion criteria for selection of paper.

Criteria type	Inclusion	Exclusion
Time period	January 2010–March 2024	–
Participants	Have been diagnosed with dementia	Mixed population including people with dementia
Concept	Employed headphones in music program	The use of headphones was not explicitly stated by authors. Loudspeaker were also used as an alternative device in music program
Context	Long term care settings (e.g., institutional, community-based, home-based facilities)	The study settings were not clearly reported
Type of sources	Type of study: Original articles, protocols, conference abstracts, project reports, students’ theses. Study design: Quantitative studies, Qualitative studies, Opinion and commentaries	Review studies Studies published not in English

### Inclusion criteria

#### Participants

The review considered studies that include residents diagnosed with all types of dementia living in LTC settings. Studies involving a mixed population including people with dementia were excluded unless the study reported the impact of headphone use explicitly among people with dementia.

#### Concept

We included studies that explicitly utilized headphones to deliver the music to residents with dementia in LTC settings. Headphones, which may be designed as over-ear, on-ear, or in-ear devices, are either connected via wires to a music device or are true wireless, offering the residents greater freedom of movement without the encumbrance of cables (JBL Human, 2024). Unlike loudspeakers that project sound into the open environment, headphones offer a private listening experience. Modern headphones which incorporate features like noise cancellation to enhance the immersive quality of the music experience were also considered. Studies that employed both headphones and loudspeakers were considered only if they distinctly analysed the impacts of headphone use.

#### Context

The studies included in this review were conducted in various LTC settings, both institutional, community-based, and home-based (Shi and Singh, 2022; National Institute on Aging, 2023). Institutional facilities typically provide comprehensive 24-h nursing care, personal care, and additional allied health services. These facilities encompass but are not limited to residential nursing homes, rehabilitation centers, intermediate care facilities, mental health facilities, hospices and hospital-based palliative care units. In contrast, community-based facilities offer care supervision and personal care by trained staff, enabling residents to maintain as much independence as possible. Examples of such settings include but not limited to home-based care services, assisted living, and day care centres. Studies that did not specify their settings were excluded from the review.

#### Types of sources

Original articles, protocols, conference abstracts, project reports, and student’s thesis published in English were considered. This scoping review considered both quantitative, qualitative and mixed-method studies. For quantitative studies, experimental and quasi-experimental study designs including randomized controlled trials, non-randomized controlled trials, before and after studies and interrupted time-series studies were considered. In addition, analytical observational studies including prospective and retrospective cohort studies, case series were considered. Qualitative study designs that were considered, but not limited to, phenomenology, grounded theory, ethnography, qualitative description, action research and feminist research. Opinion and comment papers that addressed the reflection of headphone use in music programs among residents with dementia in LTC settings were also considered. Review studies were not included in this study. However, the references of related reviews were examined by hand search.

#### Search strategy

A three-step search strategy, as recommended by the JBI review guidelines, was adopted in this scoping review. Initially, a limited search was conducted using MEDLINE (via PubMed), CINAHL (via EBSCO) and the JBI Database of Systematic Reviews and Implementation Reports databases to identify studies relevant to the topic. Then, keywords words from the titles and abstracts of these studies, along with their index terms, were used to develop full search strategies. These strategies were tailored for each database, including Embase, Web of Science, Scopus, and PsycINFO (via ProQuest) and ProQuest. The third step involved screening the reference lists of all selected articles to uncover additional relevant literature. Google Scholar searches employed combined terms related to headphone-based music programs: ‘Headphone music’ OR ‘music’ OR ‘music therapy’ OR ‘music intervention’ OR ‘individualized music’ OR ‘personalized music’, terms for dementia: ‘Dementia’ OR ‘Alzheimer’ and terms for LTC homes: ‘long-term care’ OR ‘nursing home‘OR ‘residential care ‘OR ‘care settings’ OR ‘hospital’ OR ‘assisted living’ OR ‘group homes’ OR ‘halfway houses’ OR ‘homes for the aged’. Collaboration with a university medical librarian ensured the refinement of the search strategy, capturing the essential literature. Additionally, an academic professor (LH) on the team, familiar with key literature, provided guidance for reference searches throughout the process.

#### Study selection

Following the search, all identified studies were compiled and uploaded into Covidence (Veritas Health Innovation, Melbourne, Australia), a web-based collaboration software platform that streamlines the production of literature reviews and can automatically remove duplicate entries. After a pilot test, two reviewers independently screened the titles and abstracts for assessment by referring to the inclusion criteria. Studies deemed potentially eligible were then retrieved in full text, and their citation details were recorded in a collaborative Excel spreadsheet. Each study was reviewed by two assigned reviewers, with both reviewers examining the full text independently to ensure consistency in the application of the inclusion criteria. Any discrepancies were discussed with the other team members to reach a consensus. All reasons for any excluding articles were recorded and reported.

#### Data extraction

Data from studies included in the scoping review were extracted by two independent reviewers using a data extraction tool developed by the reviewers. This tool was designed based on JBI template source of evidence details (Peters et al., 2020), characteristics and results extraction instrument, tailored to the research questions of our review. A pilot test was conducted with the extraction tool during which two reviewers independently extracted data from six and compared the results to refine the tool. Further, data extraction was carried out using the modified tool, with continuous adjustments made as necessary throughout the process. Whenever modifications were made to the data extraction, we re-reviewed all previously extracted articles to ensure consistency with the updated extraction tool. The data extracted and recorded on the final extraction table included: (1) first author name and year of publication; (2) country of study; (3) study aim and design; (4) participants (number of participants receiving headphone-based music program, types of dementia, severity of dementia, age, and gender); (5) types of LTC settings; (6) details of the music program (e.g., methods for music playlists development, type of music, setting to listen to music, dosage, length); (7) headphone details (e.g., type of headphones, other equipment used); (8) barriers and enablers explicitly in headphone use; and (9) barriers and enablers in implementing music programs. Discrepancies in data extraction were resolved through discussion among the authors.

#### Data analysis and presentation

The focus of this review was to identify and describe evidence on music programs delivered via headphones to residents with dementia, as well as to explore the associated barriers and enablers. Given that headphones and music are closely intertwined, and both play a crucial role in the program, it is essential to consider factors related to music implementation alongside those specific to headphone use. Therefore, both barriers and enablers related to both headphone use, and other aspects of music implementation were analysed and presented separately in this review.

Thematic analysis was employed to analyse the extracted data. An inductive approach was used to identify themes, which involved the authors immersing themselves into the content to highlight key concepts related to barriers and enablers that appeared to repeat across all the articles (Braun and Clarke, 2006). These initial key concepts were recorded as categories, which were then further organized and grouped into themes based on similarity (Hassan et al., 2024; Pollock et al., 2023).

## Results

**Figure 1:**
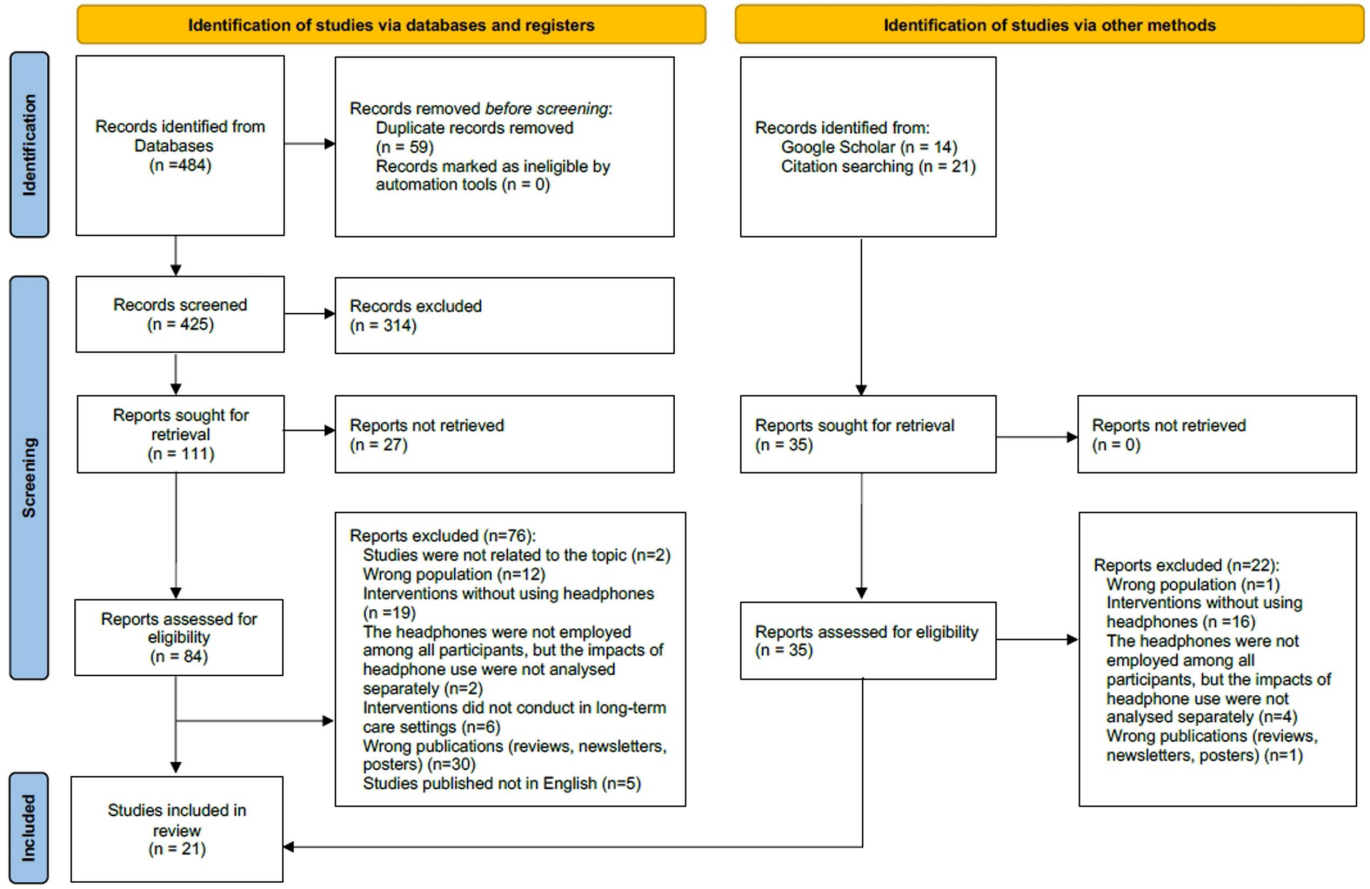
PRISMA flow diagram.

A total of 484 records were identified, from which 59 duplicates were removed. Following initial title and abstract screening against the selection criteria, a total of 314 records were removed before obtaining full text. Eighty-four full text studies were retrieved of which 21 studies were eligible. The search process refers to the PRISMA flow diagram in Figure 1.

### Music programs using headphones

Of the 21 studies included in the review, 10 studies (47.6%) were conducted in the United States, four (19%) in Australia, six studies (28.57%) in European countries, and one study in Brazil. Among the studies, 12 used quantitative approaches, two used qualitative approaches and two used mixed methods. Most of the quantitative studies were RCTs (n = 7), while the other were quasi-experimental studies featuring a before and after study design (n = 5).

Regarding headphones, most studies using headphones did not specify their characteristics, although two studies noted that over-ear type were used. Headphones were primarily connected to Apple portable music devices (n = 10), including iPod/iPod Shuffle, iPod Touch, iPhone and iPad. Other devices used included MP3 player (n = 4), traditional audiotape player (n = 3), personal digital music devices (n = 2), and notebook (n = 1). Only one study did not report the type of music devices used.

The studies involved various music programs delivered via headphones, with program durations ranging from 3 to 32 weeks, most lasting 4 and 6 weeks. The frequency of sessions varied from 1 to 5 times per week, with some studies adjusting the frequency based on residents’ signs of agitation. Music sessions typically lasted between 15 and 45 min, with 30–40 min most employed. Nearly all programs included personalized music determined by the preferences of residents and their family members, except for one study where the music list for each institution was previously defined by researchers (Corrêa et al., 2020). The Music & Memory (M&M) was the largest headphone-based music program identified in the review which has been adopted in over 1,500 LTC homes in the US. Five out of 21 studies (23.81%) were derived from M&M programs, reporting on the effectiveness and implementation of personalized music in various regions of the US, including nursing homes in Texas, Wisconsin, the Midwest, the Mid-Atlantic, and the southern regions, as well as an assisted living facility (ALF) in North Carolina (Harrison et al., 2021; Kwak et al., 2016, 2021; McCreedy et al., 2021; Murphy et al., 2018). More details on the characteristics of included studies are presented in Table 2.

**Table 2:** Characteristics of included studies (n = 21).

Study, year	Country	Aim	Study design	Participants (severity of dementia, number, age, and gender)	LTC settings (Context)	Music program (Concept)					Headphone (Concept)	
						Methods for music playlists development	Type of music	Place to listen to music	Dosage	Length	Type of headphones	Other equipment
Prick et al. (2024)	Netherlands	To reduce neuropsychiatric symptoms and enhance quality of life	RCT	56 residents with dementia from very mild, mild, moderate, severe and very severe cognitive decline; 81.8 ± 9.3 years; 76.8% female	Nursing home	1) Find out personal music preferences using a standardized inventory instrument of personal music preferences: APMPQ 2) Interviews with person with dementia and their close relatives	No restriction	Residents’ room as a quiet and comfortable environment	3 times a week on non-consecutive days during 30–45 min	3 weeks	Headphones without further description	iPod
Grainger (2024)	US	To reduce agitation and the use of anxiolytic and antipsychotic medications	Quasi-experimental study (Protocol)	10 residents with Dementia; BIMS score of <13/15	Skilled nursing facility	1) Music included preferred music from young adulthood (ages 16–26) 2) Music preferences were sought from family recommendations, or Top 40 songs from young adulthood	No restriction	Facility’s activities room	Twice a week for week 1–4 and 4 times a week for week 5–6 during 30 min	6 weeks	Headphones without further description	MP3 players
Hillebrand et al. (2023)	Germany	To reduce BPSD of dementia	RCT	61 residents with Advanced/severe dementia	Nursing homes	1) Find out personal music preferences using a list of examples of popular artists and song titles; 2) Gather information from family members, nursing home staff, and directly from participants if they are able to verbalize their personal preferences; 3) Interviews from family members and/or participants	No restriction	NR	Every second day for 20 min	6 weeks	Headphones without further description	MP3 players
Gulwani (2022)	US	To reduce agitation, aggressive behaviors and use of psychotropic medications	Quasi-experimental study	10 people with dementia with BIMS mean 6.2 ± 4.7; 80 ± 10.9 years; 50% female	Nursing home	Asking resident’s and family’s preferences through phone calls. A preferred music list was developed for each resident.	No restriction	NR	Twice a week (Tuesday and Thursday) for 30 min	6 weeks	Headphones without further description	MP3 players, storage box, disinfectant.
Gaviola et al. (2022)	Australia	To explore the experiences and perceptions of using individualized music	Qualitative study	32 people with Alzheimer’s-type dementia; Severe to very severe stage; median age of 86 year; 23 females and 9 males.	Residential aged care facilities	Created with each older participant and his or her family member	No restriction	A location where the person spends most of his or her time	Suggested duration is 30 min, however, music may be played for as long as older person enjoys it as assessed	NR	Padded headphones	iPod Shuffle
Amor Gaviola et al. (2022)	Australia	To determine the cost of implementing an individualized music intervention	Cost analysis	32 residents with dementia	Residential aged care facilities	About 30-min interview with resident with dementia or their family or guardian about music preferences	No restriction	NR	30 min per session, the frequency depends on residents’ wishes or needs	12 weeks	Headphones without further description	iPod Shuffle
McCreedy et al. (2021)	US	To reduce agitated behaviors	RCT (Protocol)	Residents with moderate to severe dementia	Nursing homes	Two strategies will be tested separately. 1) Activity staff identify 25–50 songs that the resident appears to like, and then tested songs with residents to look for a positive reaction 2) Research staff preload music players based on the demographics of the resident and his/her preferred genre (if known)	Beloved songs from a person’s formative years; tap deep memories long attached to the brain	NR	Recommend 30 min per day; times of day when behaviors were likely or at early signs of agitation	8 months	Earphones without further description	Personal music devices
Lumsden (2021)	US	To reduce dementia-related agitation	Quasi-experimental study	4 people with dementia, advanced age, female	Memory care unit in a long-term care facility	Family member responses recorded on the music preference form (favorite genre, time period, bands, and musicians)	No restriction	Either the namaste room. Common area, or residents’ room	At least 30 min or until the episode of agitation is resolved	3 months	Headphones without further description	iPhone and iPad
Kwak et al. (2021)	US	To examine enablers and barriers related to the implementation and sustainability of the Music & Memory program	Mixed methods	People with dementia	Nursing homes	Care staff set up personalized music playlist for residents based Selected based on resident’s autobiographical memory and musical preferences	No restriction	NR	An average of 1–5 times per week	NR	Headphones without further description	iPods, iPod Shuffles, non-Apple portable music players, and computers
Kuot et al. (2021)	Australia	To evaluate how integrating personalized digital music playlists would influence behaviors, well-being and clinical management	Qualitative	10 residents with dementia with moderate or advanced dementia; mean 81 years	Rural aged-care home	1) Residents’ families assisted in providing the lists of songs the residents used to like, played or listened to before the onset of dementia 2) The playlists subsequently revised throughout the 8-week intervention according to 5 key strategies: ① preferred genres noted by family or observation of staff during music related activities; ② era-specific music according to resident’s date of birth; ③ experimentation around the above 2 strategies using iTunes libraries of digital music playlists; ④ minor revision or abandoning of certain song in the created music playlists if negative responses are observed	No restriction	NR	30 min per day	Total 60 sessions 8 weeks	Headphones without further description	iPod
Harrison et al. (2021)	US	To reduce agitation, control cognitive impairment	RCT	162 people with dementia, Mean Score of MMSE: 8.4 ± 8.8; 79.7 ± 11.2 years; 71.6% females	Nursing homes	The family, resident, and staff assented to work together to identify music for participants selected from an electronic list to be stored on personal digital devices.	No restriction	NR	NR	8 weeks	Headphones without further description	Water proof personal digital device used to store music
Corrêa et al. (2020)	Brazil	To compare the physiological, behavioral and expressive effects of Brazilian popular songs and classical music	Quasi-experimental	33 older adults with severe dementia; 85.1 ± 8.68 years and 89.5% females for popular music group vs. 85.3 ± 7.6 years and 71.4% females for classic music group	Long-term care institutions for the elderly (ILPI)	Previously defined for each institution	Brazilian Popular music and classic music	Suitable and silent room, previously prepared.	20 min per session, 4 sessions, once a week	4 weeks	A Sony Headphone with dimensions 207x57x271mm (AxLxP), of the circumaural or over-ear type (that sits around the ear)	Notebook, Cardio emotion and Non-invasive sensors
Weise et al. (2019)	Germany	To evaluate the feasibility and effects of an individualized recorded music listening intervention on the BPSD	RCT	20 residents with mild to severe dementia; 85.05 ± 5.93 years; 80% females	Nursing home	Social service staff and project staff used questionnaires and interviews (telephone or face-to-face) to identify personally-relevant music for each participant Gathered information from family members, nursing staff and directly from participants if they were able to verbalize their preferences. Compiled up to 3 individualized playlists for each participant. The suitability of the playlists was checked during the first sessions and playlists were continuously adapted over the intervention period as needed.	No restriction	NR	30 min every other afternoon	4 weeks, total 14 sessions	Headphones without further description	MP3 players
Ihara et al. (2019)	US	To examine the effects of a person-centered music listening intervention on mood, agitation and social engagement	Quasi-experimental study	31 residents with dementia	Adult day health centers	Ask caregivers about the participant’s favorite music or by playing different songs for participants to see their reactions.	No restriction	A comfortable room could seat 7–10 people with temperature appropriate for the season. The door was closed and only the researchers and participants were present in the room.	20 min twice a week	6 weeks	Headphones without further description	iPod
Murphy et al. (2018)	US	To evaluate the implementation of a personalized music listening program, focusing on its reach, effectiveness, adoption, implementation and maintenance	Mixed-methods study	17 residents with dementia	Assisted living facility	Interviews with residents and their family members guided by a simple music preference form; Personal CDs belonging to residents or family members were used to develop individual playlists Playlists were adjusted based on resident response to music on a bi-monthly basis.	No restriction	NR	Sessions were conducted regularly, but the specific frequency is not detailed in the document. The duration of each listening period was approximately 30 min.	8 months	Over-the-ear headphones	iPod Shuffles
Shiltz et al. (2016)	US	To determine how personalized music delivered via headphones influenced affect, behavior and cognition/memory as well as the BPSD, thereby affecting the necessity and utilization of pharmacological interventions for agitation.	RCT	92 residents with mild, moderate or severe dementia	Extended care facility	Researchers along with undergraduate music students and psychology majors met with participants and their families to determine the subject’s preferred musical genre and specific preferred songs from their late teens to early twenties.	No restriction	NR	30 min 3 times a week	3 months	Headphones without further description	iPod Shuffles
Kwak et al. (2016)	US	To provide information on the methods and findings from all four components of the Music and Memory program evaluation	Project Report	Residents with moderate to advanced dementia were enrolled; 59 residents participated in RCT (Study 1); 1,500 residents in quasi-experimental study (Study 2); A large number of residents participated in the implementation study	Nursing homes	Identify individual’s music preferences and songs significant to that person’s life experience from persons with dementia or family members; In case a person is no longer communicative and family members have little information about their relative’s music preferences, caregivers can play music popular from the time the individual was a child or young adult, including music played on the radio or in popular television shows, and judge what is preferred based on the individual’s reaction to the music	No restriction	No restriction	NR	14 weeks for study 1 NR the length for other studies	Headphones without further description	iPhone or iPod; an app installed on the iPod Touch to track the use of music
Locke and Mudford (2010)	New Zealand	To assess empirically the observed (anecdotal) effect music had on the chanting and speech-like vocalizations of a man with dementia.	Case report	A 68-year-old Thai man with early onset Alzheimer’s dementia for 6 years	A secure dementia unit of a rest home and hospital facility	Residents’ family advised on his preferred music genres	A variety of popular music from the 1950s and 1960s was played, along with some classical music	No restriction	Four 5-min conditions: Baseline (no music or headphones), Ambient Music (music played from an audiotape player without headphones), Music-via-headphones, and Headphones only (no music). 10 once-daily 20 min sessions of music via headphones were implemented after the assessment phase. Five once-weekly 20 min follow up treatments were conducted as a follow-up.	6 weeks	Sony headphones	A small Transonic audiotape player 12 cm x 7 cm x 2 cm and a portable Transonic Discman
Guetin et al. (2009)	France	To assess the effects of this new music therapy technique on anxiety and depression in patients with mild to moderate Alzheimer-type dementia.	RCT	15 residents with mild to moderate stages of Alzheimer’s disease; mean 85.2 ± 6 years; 86.7% females	Nursing home	The music was chosen based on the patients’ personal tastes following an interview/ questionnaire. Choosing music connected to the individual’s personal experience is of paramount importance. A computer program makes it possible to select a musical sequence suited to the patient’s request from the different musical style suggested; The standard musical sequence, lasting 20 min, is broken down into several phases which gradually bring the patient into a state of relaxation according to the ‘U sequence’ methods, specially created by the record publishing company, Music Care.	Classical music, jazz, world music, various	In patients’ rooms.	Once a week, lasting 20 min	16 weeks	Headphones without further description	Mask
Garland et al. (2007)	Australia	To compare the effectiveness of two individualized psychosocial treatments in reducing the frequency of physically and verbally agitated behaviors in nursing home residents whose dementia was complicated by marked behavioral disturbance	RCT	30 residents with very low mean MMSE score: 2.5; Mean age 79 years; 63% females	Nursing homes	Music selections were based on family members’ reports of subjects’ preferences	No restriction	Popular songs, big band music, Greek and Dutch music	Each session was conducted once a day for 3 days during each treatment week. Each session lasted for 15 min.	4 weeks (Week 1 involved usual care observations, Week 2–4 involved treatments and music delivery)	Headphones without further description	Portable cassette
Ragneskog et al. (2001)	Sweden	To find out if individualized music reduces agitation and leads to emotional reaction.	Quasi-experimental study	1 patient (George) with sever dementia; 77 years old	Nursing home	Selected after discussion with the patients, the patients’ next of kin and the nursing staff.	Operetta, Ballads: Lasse Dahlqvist	Sat in the sitting room in a chair with a fixed table	45 min per session, and 69 sessions in total	NR	Headphones without further description	Cassette tape recorder

APMPQ, Assessment of Personal Music Preference Questionnaire; BIMS, Brief Interview for Mental Status; BPSD, Behavioral and psychological symptoms.

**Figure 2:**
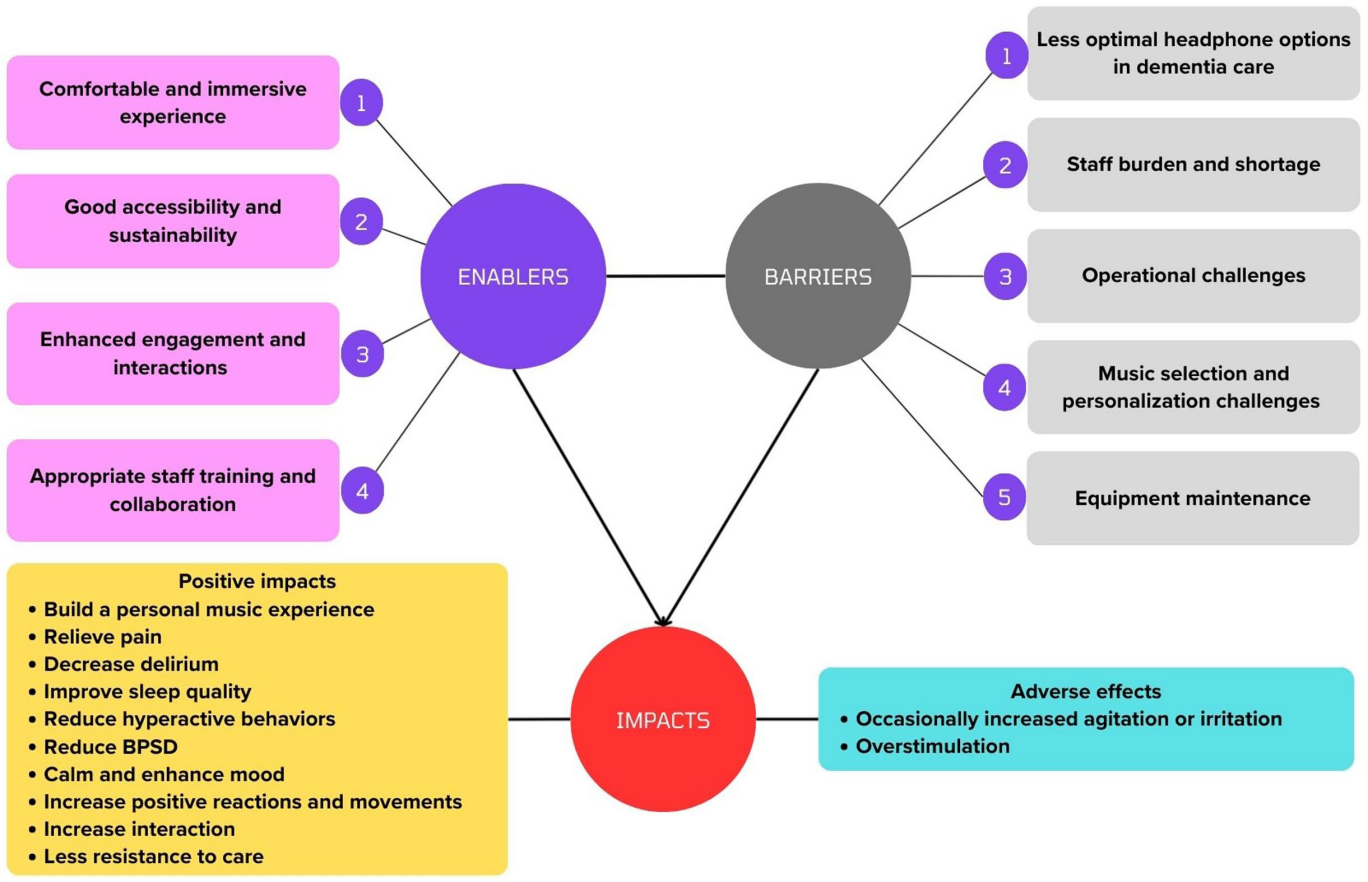
A visual summary of the final themes.

Authors of only three studies had the primary goal of identifying barriers and enablers to the headphone use in music intervention, all of which were M&M conducted in nursing homes and ALF (Kwak et al., 2016, 2021; Murphy et al., 2018). Barriers and enablers in these studies were identified from authors’ reflection in results and discussion. See Figure 2 for the final themes, with more detailed information available in Supplementary Tables S1 and S2.

### Enablers for using headphones in music programs for people with dementia in LTC settings

#### Comfortable and immersive experience

Comfortable experience first involves ensuring that the residents with dementia are suitable candidates for headphone use, as some individuals may not tolerate the headphones well. Several studies specifically included those residents who could comfortably use headphones (Harrison et al., 2021), had normal hearing ability through headphones (Gulwani, 2022; Harrison et al., 2021; Shiltz et al., 2016), did not wear hearing aids (Guetin et al., 2009), or could wear headphones for at least three consecutive sessions of the intervention (Garland et al., 2007).

Verifying that the headphone volume is set to a safe and comfortable level before the music begins is another step to ensure that the headphones are not only suitable for the resident’s physical needs but also for their sensory comfort during the intervention (Corrêa et al., 2020; Locke and Mudford, 2010; Prick et al., 2024). In particular, a volume of 60–70 decibels, equivalent to that of normal conversation, was reported as appropriate for headphone use (Corrêa et al., 2020).

Creating an adequate environment is an especially important enabler for enhancing the comfortable and immersive experience of listening music using headphones. An adequate music environment is characterized by comfort, familiarity, and quietness, which facilitates the music experience and minimize distractions. Several studies were conducted in silent rooms that were familiar to the residents (Corrêa et al., 2020; Prick et al., 2024), equipped with comfortable seats (Guetin et al., 2009; Ihara et al., 2019), maintained at an appropriate temperature for the season (Ihara et al., 2019), and restricted to researchers and music listeners only (Ihara et al., 2019). To help residents with dementia fully immerse in the music experience, they should be allowed to choose a comfortable position and provided with a mask to minimize visual stimuli (Guetin et al., 2009).

#### Good accessibility and sustainability

One of the major themes that came through was around the accessibility and sustainability of headphones. The ease of access to headphones in LTC settings makes their use for delivering music programs highly feasible. Studies indicated that headphones can be conveniently placed in resident rooms, on the unit, or in open areas for immediate access and continuous availability, 24/7 (Kwak et al., 2021). Moreover, Sustainability is also a significant enabler, with reports highlighting that headphones can be “well stored,” “reused” and are characterized by “low cost of buy-in” (Amor Gaviola et al., 2022; Grainger, 2024; Gulwani, 2022; Hillebrand et al., 2023; Kwak et al., 2016; Murphy et al., 2018). Additionally, financial support and donations, as noted in three studies, played a crucial role in the long-term sustainability of headphone use, facilitating the ongoing maintenance of headphone-based music programs in LTC settings (Kwak et al., 2016, 2021; Lumsden, 2021).

Moreover, the straightforward nature of using headphones in music programs contributed to its accessibility and sustainability as an enabler in LTC settings. Listening to preferred music via headphones required less expertise and resources compared to more complex behavioral procedures (Locke and Mudford, 2010), which can be conducted by nursing and direct care workers as part of their daily care regime (Kuot et al., 2021). The intervention did not necessitate a license (Amor Gaviola et al., 2022), was safe with minimal adverse effects (Gulwani, 2022), making it a practical and accessible option for enhancing engagement with music among residents with dementia.

#### Enhanced engagement and interactions

Immediate positive responses and personalized sensory input influenced residents’ engagement. Headphones enable residents with dementia to experience music in a highly personalized manner, eliminating restrictions related to time, space, and posture (Locke and Mudford, 2010). This flexibility enables personalized auditory stimulation, allowing residents to enjoy their preferred music at any moment without concern for other’s music tastes or the risk of disturbing other residents in LTC settings. Such individualized music delivery via headphones has been shown to enhance residents’ positive responsiveness to music, thereby fostering greater engagement from both family members and staff (Gaviola et al., 2022; Locke and Mudford, 2010; Garland et al., 2007; Murphy et al., 2018).

Of note, headphones did not hinder social interactions. Even if others cannot hear the music directly, it still can be shared through immediate responses, such as dance and physical vibration, providing an alternative form of interactive engagement (Harrison et al., 2021). This combination of personalized listening and potential for social interaction highlights the powerful role of headphones in music programs for residents with dementia.

#### Appropriate staff training and collaboration

Staff training and collaboration were identified as key implementation enablers in 12 studies. Due to COVID-19 constraints and shifting duty schedules, training failed to reach all staff members, which affected the consistency of the intervention (Lumsden, 2021). Training encompassed specific tasks related to headphone use, including proper distribution, setup, and facilitation of music listening via headphones, as well as monitoring residents’ reactions and collecting the headphones after the session (Harrison et al., 2021; Kuot et al., 2021; Murphy et al., 2018). To ensure continuous headphone use during the music sessions, staff were also required to monitor usage closely, reminding residents to keep their headphones on and assisting those who might remove them during listening (Grainger, 2024; Gulwani, 2022). In addition to task-specific training, most studies emphasized the importance of comprehensive training for implementing the entire music program using headphones. This involved educating research teams, staff, relatives, and informal carers about the broader goals and structure of the program (Amor Gaviola et al., 2022; Corrêa et al., 2020; Gaviola et al., 2022; Gulwani, 2022; Harrison et al., 2021; Lumsden, 2021; Weise et al., 2019). Training typically included instruction on administering the intervention, housing equipment, and recognizing signs of discomfort (Corrêa et al., 2020; Harrison et al., 2021).

Collaboration was also emphasized, with studies noting the positive involvement of facility personnel in program initiation (Kwak et al., 2016; Murphy et al., 2018; Weise et al., 2019). In two studies, LTC staff administered the intervention with support from research staff (Kwak et al., 2016, 2021), while activity staff were responsible for implementing and monitor participants’ reactions to the music in another two studies (Prick et al., 2024; Weise et al., 2019). Moreover, in some studies, relatives, informal caregivers and volunteers received training and were involved in delivering the music intervention during their visit (Amor Gaviola et al., 2022; Kwak et al., 2016, 2021; Murphy et al., 2018).

### Barriers of using headphones in music programs for people with dementia in LTC settings

#### Less optimal headphone options in dementia care

The suitability of available headphones was a key concern as residents with dementia were often older adults with varying degrees of hearing impairment (Hillebrand et al., 2023). Traditional in-ear headphones might be unsuitable, particularly for those using hearing aids (Kwak et al., 2021; Murphy et al., 2018). Some headphones may fail to adequately isolate music from background noise or self-generated sounds (e.g., vocalizations) as one study observed residents can still interact with one another and/or researchers while listening (Ihara et al., 2019).

Furthermore, residents with dementia sometimes face challenges in adapting to using existing types of headphones. Even those who were physically able to use headphones, several simply did not like to wear headphones or refused them occasionally after trying (Garland et al., 2007; Kuot et al., 2021; Kwak et al., 2016, 2021). Although some headphones were padded for comfort, the pressure and size of headphones can still cause discomfort during prolonged use (Gaviola et al., 2022).

#### Staff burden and shortage

The use of headphones in music programs for residents with dementia in LTC homes required ongoing staff support. Even where the headphone was easy to use, staff were expected to spend extra time and effort for tasks such as identifying the music preferences, allocating music equipment, continuous monitoring and ensuring devices were charged (Amor Gaviola et al., 2022; Gaviola et al., 2022; Murphy et al., 2018). Moreover, music equipment connected with headphones, such as iPods, was often not user friendly for senior residents with dementia, leading to frustration and frequent requests for assistance from staff to operate the device and control the volume (Kwak et al., 2016, 2021). Several studies noted that existing staff were already overburdened by the multiple roles, further complicating the implementation of a headphone-based music program (Harrison et al., 2021).

Particularly, regarding the headphone use, staff need to deliver and install headphones, adjust the volume, turn on the music, check residents’ position, and manage the removal, clean and storage of headphones (Amor Gaviola et al., 2022). Occasionally, some residents with dementia struggled to remove headphones independently, requiring more assistances during collection (Kwak et al., 2016). Residents with moderate to severe dementia who exhibited agitation needed extra attention from staff to keep headphones in place or to prevent falls, especially when they had increased desire to stand and dance during music sessions (Murphy et al., 2018). Therefore, even with direct care staff assigned to handle these tasks on a set schedule, the added responsibilities could create a burden (Kwak et al., 2016). Without direct care staff buy-in, implementation would take even longer (Kwak et al., 2021).

In fact, the lack of staffing extended the barrier beyond headphone management to the broader issue of lack of human resources needed to run the entire music programs. This shortage not only included care staff but also supportive family members and volunteers (Kwak et al., 2016, 2021). Additionally, high turnover rates among staff and volunteer resulted in the needs for continuous training and renegotiating of logistics (Gulwani, 2022; Kuot et al., 2021; Kwak et al., 2021; Murphy et al., 2018; Prick et al., 2024).

#### Operational challenges

Operational challenges in the use of headphones primarily involve concerns about the decline in headphone usage and difficulties in tracking the timing and frequency of use. When headphones were provided only upon request or needed, their use can decline, especially among residents with cognitive deficits who struggle to request them (Murphy et al., 2018). Additionally, making headphones available to residents whenever they are awake poses challenges for staff in tracking when and how often they are being used (Kwak et al., 2016). From music therapists’ perspective, they were concerned that LTC facilities might perceive headphones as simple replacement for their specialized work, particularly if implementation was solely managed by nursing staff. This perception could devalue the role of music therapists in providing personalized, therapeutic interventions (Kwak et al., 2016, 2021).

#### Music selection and personalization challenges

A lack of appropriate music selection and personalization was presented as a barrier to the implementation of the headphone-based music programs. Some residents initially refused music or exhibited withdrawn moods, often due to poor initial music selection or playlists that did not align with their preference (Murphy et al., 2018). In some cases, families were not supportive or helpful in music playlist development and selection of playlists (Kwak et al., 2016), and residents with dementia did not enjoy the music recommended by their family members (Murphy et al., 2018). Of note, residents’ musical preferences and tastes can change over time, making it difficult to maintain engagement with the same playlist (Kuot et al., 2021; Murphy et al., 2018). However, allocating extra budget for buying new songs added a financial burden to the program (Kwak et al., 2016, 2021; Murphy et al., 2018). Moreover, some residents preferred quiet environment or were simply not interested in music, further making it harder to personalize the intervention to meet their needs (Kwak et al., 2016, 2021).

#### Equipment maintenance

Concerns about equipment maintenance focused on challenges associated with managing and ensuring the consistent use of music equipment. Key issues included difficulties with proper storage, turning off and timely charging of devices. Staff expressed frustration when equipment was not properly stored or having problems with charging, and sometimes devices were lost and never returned to storage (Gaviola et al., 2022; Kwak et al., 2016, 2021; Murphy et al., 2018; Weise et al., 2019). The small size of devices like iPod and MP3 players increases their risk of being misplaced, and without GPS tracking, locating, and retrieving them became difficult (Kwak et al., 2016; Murphy et al., 2018). Furthermore, iPod Shuffles were no longer available in the market, as more expensive iPod Nanos had to be used to maintain established music libraries, resulting in extra budget (Murphy et al., 2018). While many studies have indicated that the cost of headphones is relatively low, ongoing financial support was still needed (Lumsden, 2021). The lack of funding for purchasing replacement headphones, due to loss or damage, could hinder the continued use of headphones for residents with dementia in LTC settings (Kwak et al., 2016, 2021; Murphy et al., 2018).

## Discussion

This scoping review is the first to systematically identify the enablers and barriers to implementing headphone-based music programs for people with dementia in LTC homes and provide actionable recommendations to guide future program design and clinical practice. Key enablers include user comfort and immersive experience, ease of access, staff training, and enhanced engagement and interactions. In contrast, less optimal headphone options, staff burden, operational challenges and difficulties with music personalization and equipment maintenance were identified as major barriers. These findings reveal the practical complexity behind implementation and highlight the need for tailored strategies to optimize headphone-based music program for residents with dementia living in LTC settings.

### Optimizing headphone fit, sound parameters, and listening contexts

Selecting appropriate headphones is essential for ensuring comfort and sustained engagement among residents with dementia. Existing evidence demonstrated that people who prefer and can tolerate headphones may benefit more from music program (Murphy et al., 2018). However, many headphone types used in studies were not compatible with hearing aids or caused discomfort due to pressure and size, even when padded (Gaviola et al., 2022; Ihara et al., 2019; Locke and Mudford, 2010). In-ear headphones were particularly unsuitable for individuals with hearing impairments or those wearing hearing aids (Guetin et al., 2009; Hillebrand et al., 2023).

Facilities should prioritize selecting dementia-friendly headphone models that are lightweight, adjustable and over-the-ear in style, which tend to be more compatible with hearing aids and reduce refusals rates (Murphy et al., 2018). Headphone features, such as super-soft ear cups, stereo sound, and foldable designs may enhance comfort and usability for residents with dementia, and have been recommended by the New York Alzheimer’s Store.^1^ In addition, residents often dropped or removed headphones due to agitation or movement, requiring more secure or fixed designs to minimize staff burden (Garland et al., 2007). A more person-centred design approach—such as on-site observation, user interviews, and co-design activities—may help address sources of discomfort and improve usability (Hassling et al., 2005).

In addition to physical fit, attention must be paid to sound-related factors such as volume and duration of headphone use. Studies reported that older adults with dementia often had difficulty adapting to long music sessions lasting 30–40 min. Although three studies mentioned adjusting volume to a comfortable level (Corrêa et al., 2020; Locke and Mudford, 2010; Prick et al., 2024), only one study specified the use of 60–70 decibels, equivalent to normal conversation (Corrêa et al., 2020). According to NIOSH guidelines, exposure to sound above 100 decibels should not exceed 15 min (Occupational Safety and Health Administration, 2023). Long-term use may also impact hearing health (Himanshi et al., 2018). Therefore, appropriate volume and session duration must be carefully determined to ensure sensory comfort and safety.

The listening context also influences adherence to headphone use. One study noted that while some residents experienced reduced anxiety when listening to music in noisy communal spaces, others required a quiet and isolated setting for full immersion (Garrido et al., 2020). To maximize benefits, staff should tailor the timing and conditions of headphone use based on individual preferences, time of day, physical or emotional discomfort, and environmental variables such as noise level and temperature. Allowing residents to listen in a comfortable position, using visual masks to reduce distractions, and ensuring appropriate ambient conditions can further enhance music engagement (Guetin et al., 2009; Ihara et al., 2019).

### Establishing standard operating protocol for headphone management

Viewed as a new technology in LTC homes, headphones have yet to be fully integrated into routine workflows. Staff highlighted the need for convenient placement of headphones and music devices, allowing for quick access without the need to locate misplaced equipment or deal with uncharged devices (Gaviola et al., 2022; Hung et al., 2024; Kwak et al., 2021). Therefore, establishing standard operating protocol for the daily management of equipment, such as assigning responsibilities for charging, cleaning, and securely storing devices can help minimize operational barriers. Then, facilitates should prioritize structured staff training that covers the proper use, handling, cleaning, and storage of headphones. This training should emphasize practical skills to build staff confidence on managing the headphones efficiently while ensuring residents have consistent and timely access to music programs (Hung et al., 2021, 2024; Kwak et al., 2016).

### Defining roles and responsibilities within a collaborative team

Team collaboration plays a vital role in effective headphone management. The included studies support recommendations for establishing clear collaborative frameworks that define roles and responsibilities across teams (Hung et al., 2024), which can help streamline processes and promote the sustainable integration of headphone-based music programs into LTC care routines Specifically, activity staff could be responsible for delivering music program (McCreedy et al., 2021), medical technicians should manage equipment storage and maintainance (Murphy et al., 2018), relatives and informal caregivers can assist residents in using headphones during their visit (Amor Gaviola et al., 2022), and research teams can play a continued role by providing training and support to new staff and volunteers (Kwak et al., 2016, 2021; Murphy et al., 2018). Furthermore, the positive involvement of music therapists can strengthen staff training and improve the quality and therapeutic value of headphone-based music programs,

To address barriers such as high staff turnover, it is important to consider strategies that foster sustained motivation (Pimentel et al., 2020). A recent qualitative study indicated that nurses were more engaged when they could see their achievement were quantified, “bite-sized” intervention formats, and when incentives were provided for their contribution. The program “champions” may also help maintain engagement by offering ongoing encouragement and peer support (Mills et al., 2019). However, motivational strategies were not identified in the implementation process reviewed in this study. Future implementations should incorporate motivation components while defining team responsibilities to improve consistency and sustainability of the headphone-based music program in LTC settings.

### Personalizing music delivery and enhancing therapeutic engagement

It is important to acknowledge that the use of headphones for music delivery should not be reduced to simply placing them on residents with dementia and playing a pre-set playlist (Kwak et al., 2016). Rather, such interventions must be implemented as part of a structured and individualized program that considers the person’s musical history, preferences, and emotional needs (Odell-Miller et al., 2021).

Several studies have pointed out that music selection and personalization remain key challenges in this context. Residents with moderate to severe dementia often have difficulty communicating their preferences, and playlists based solely on family interviews may not fully reflect the resident’s actual music tastes. This misalignment can limit engagement with the intervention and reduce adherence to headphone use (Murphy et al., 2018). To address this, facilities are encouraged to incorporate life story work or reminiscence-based approaches to identify music that holds personal meaning and can evoke emotionally significant memories (Hillebrand et al., 2023).

In addition, electroencephalography (EEG) biomarkers or heart rate variability combined with machine learning are promising in developing bio-personalized music recommendation systems (Adamos et al., 2016; Chiu and Ko, 2017). These approaches allow for real-time monitoring of emotional and physiological responses to music by measuring autonomic nervous system activity (Chang et al., 2017; Marsman et al., 2024). Based on such data, specific musical elements—including timbre, tempo, rhythm, pitch, and harmony—can be dynamically adjusted to better align with the listener’s current emotional state and therapeutic goals (Adamos et al., 2016; Chiu and Ko, 2017). While using these technologies may not be immediately feasible in many LTC settings, they represent promising future directions for research and innovation. In collaboration with research or technology partners, such approaches could eventually support the development of low-burden, adaptive music recommendation systems tailored to the emotional and physiological states of residents with dementia.

Another practical consideration is the need to maintain listener engagement over time. Residents may become less responsive to repeated exposure to the same music. To mitigate this, several studies recommend updating playlists on a regular basis—for example, by incorporating new selections from the same artist, album, or genre (Murphy et al., 2018; Weise et al., 2019). Beyond the technical delivery, it is also important to clearly communicate to residents and their families that music listening through headphones serves a therapeutic purpose. Framing the intervention in this way can promote greater understanding and acceptance and may help reinforce its integration into routine care.

### Limitations

Several limitations need to be considered when interpreting the findings of this scoping review. First, the included studies varied in design, sample size, doses and length of the music intervention. The summarized effectiveness of the music programs using headphones compared to loudspeakers or other devices needs to be taken with caution and warrants further investigation. Additionally, some studies did not provide detailed descriptions of the types and characteristics of headphones used, making it challenging to determine which kind of headphones are most suitable for people with dementia in LTC homes.

## Conclusion

This scoping review provides a comprehensive summary of the evidence on using of headphones for the delivery of music programs for people with dementia in LTC homes. The scoping review identifies key enablers for using headphones, such as comfortable and immersive experience, good accessibility and sustainability, enhanced engagement and interactions, and appropriate staff training and collaboration. Barriers included less optimal headphone options in dementia care, requirement of staff support, operational challenges, and music selection and personalization challenges. Future headphone-based music programs should prioritize identifying the optimal headphone models and settings (e.g., volume, duration), tailoring timing and conditions for headphone use, establishing standard operating protocol, clear defining team roles, as well as educating residents and families on the therapeutic purpose of music programs. Implementation research is warranted to evaluate both the process and effectiveness of these strategies in real-world LTC settings.

## Data Availability

The original contributions presented in the study are included in the article/Supplementary material, further inquiries can be directed to the corresponding author/s.

## Footnotes

1
https://www.alzstore.com/headphones-p/2118.html
