# Supplementary material for: Use of headphones for the delivery of music programs for people with dementia in long-term care homes: a scoping review
Source: Front Dement. 2026 Jan 16;4:1707201. doi: 10.3389/frdem.2025.1707201 (PMC12857178; doi:10.3389/frdem.2025.1707201)
Supplement: Supplementary file 1 [file Table_1.docx]

**Table S1. Most frequently described enablers and barriers to deliver music programs using headphones for people with dementia in long-term care homes.**

| **Domains** | **Themes** | **Barriers/enablers described in articles** | **Number of articles, n/21 (%)** |
| --- | --- | --- | --- |
| **Enablers for headphone use in music program** | Comfortable and immersive experience | Ability to hear and tolerate headphones (Garland et al., 2007; Guetin et al., 2009; Gulwani, 2022; Harrison et al., 2021; Shiltz et al., 2016)  User sensory comfort: comfortable volume (Corrêa et al., 2020; Locke & Mudford, 2010; Prick et al., 2024)  Comfortable position (Guetin et al., 2009)  Comfortable room (Corrêa et al., 2020; Ihara et al., 2019; Prick et al., 2024)  Immersive stimulation (Guetin et al., 2009)  Monitor headphones during the music sessions (Grainger, 2024; Gulwani, 2022) | 11 (52.4%) |
|  | Good Accessibility and sustainability | Easy to access (Kwak et al., 2016; Kwak et al., 2021)  Easy to store and reuse (Kwak et al., 2016; Murphy et al., 2018)  Low cost of buy-in (Amor Gaviola et al., 2022; Grainger, 2024; Gulwani, 2022; Hillebrand et al., 2023)  Financial support (Kwak et al., 2016; Kwak et al., 2021; Lumsden, 2021)  Straightforward nature: required less expertise and resources, and had few adverse effect (Amor Gaviola et al., 2022; Gulwani, 2022; Hillebrand et al., 2023; Kuot et al., 2021; Locke & Mudford, 2010) | 10 (47.6%) |
|  | Enhanced engagement and interactions | Increased positive responsiveness (Murphy et al., 2018; Gaviola et al., 2022)  Flexible movement while listening (Locke & Mudford, 2010)  Listening without disturbing others (Locke & Mudford, 2010; Murphy et al., 2018)  Personalized sensory input (Garland et al., 2007)  Shared experience through dance and vibration (Harrison et al., 2021) | 6 (28.6%) |
|  | Appropriate staff training and collaboration | Trained on completing specific tasks related to headphones (Harrison et al., 2021; Kuot et al., 2021; Murphy et al., 2018)  Staff training on music implementation (Amor Gaviola et al., 2022; Corrêa et al., 2020; Gaviola et al., 2022; Gulwani, 2022; Harrison et al., 2021; Kwak et al., 2016; Kwak et al., 2021; Lumsden, 2021; Murphy et al., 2018; Weise et al., 2019)  Collaboration: facility administrator, medical technicians, nurses, nursing assistants, activity staff, relatives, informal caregivers and volunteers (Kwak et al., 2016; Kwak et al., 2021; Lumsden, 2021; Murphy et al., 2018; Prick et al., 2024; Weise et al., 2019) | 12 (57.1%) |
| **Barriers of using headphones in music programs for people with dementia in LTC settings** | Less optimal headphones options in dementia care | Suitability concerns (Hillebrand et al., 2023; Kwak et al., 2021; Murphy et al., 2018)  Ineffective at blocking noise (Ihara et al., 2019; Locke & Mudford, 2010)  Hard to adapt to wearing headphones (Garland et al., 2007; Gaviola et al., 2022; Kuot et al., 2021; Kwak et al., 2016; Kwak et al., 2021)  Discomfort from prolonged use (Gaviola et al., 2022) | 8 (38.1%) |
|  | Staff burden and shortage | Required more staff time (Amor Gaviola et al., 2022; Kwak et al., 2016)  Required more staff effort (Amor Gaviola et al., 2022; Gaviola et al., 2022; Harrison et al., 2021; Kwak et al., 2016; Kwak et al., 2021; Murphy et al., 2018)  Lack of human resources for implementing the music programs (Kwak et al., 2016; Kwak et al., 2021)  High staff and volunteer turnover rate (Gulwani, 2022; Kuot et al., 2021; Kwak et al., 2021; Murphy et al., 2018; Prick et al., 2024) | 9 (42.9%) |
|  | Operational challenges | Providing headphones upon request led to declined usage (Murphy et al., 2018)  Difficulties in tracking headphone usage (Kwak et al., 2016)  Concerns about devaluing the role of music therapist (Kwak et al., 2016; Kwak et al., 2021) | 3 (14.3%) |
|  | Music selection and personalization challenges | The initial music playlists did not align with their preference (Kwak et al., 2016; Murphy et al., 2018)  Music tastes changed over time (Kuot et al., 2021; Kwak et al., 2016; Kwak et al., 2021; Murphy et al., 2018)  Simply not interested in music (Kwak et al., 2016; Kwak et al., 2021) | 4 (19.0%) |
|  | Equipment maintenance | Having problems in charging: battery dead, difficult to keep the devices charged (Gaviola et al., 2022; Kwak et al., 2016; Kwak et al., 2021; Murphy et al., 2018; Weise et al., 2019)  Having problems in storage: can be lost due to small size, difficult to locate, and collect (Gaviola et al., 2022; Kwak et al., 2016; Murphy et al., 2018)  Products upgrade: have to replace iPod Shuffles with iPod Nanos (Murphy et al., 2018)  Additional cost for purchasing more headphones (Kwak et al., 2016; Kwak et al., 2021; Lumsden, 2021; Murphy et al., 2018) | 6 (28.6%) |

**Table S2 Reported barriers and enablers in using headphones in music programs among people with dementia**

| **Study, year** | **Barriers explicitly in headphone use** | **Other barriers in implementing music programs** | **Enablers explicitly in headphone use** | **Other enablers in implementing music programs** | **Impact** |
| --- | --- | --- | --- | --- | --- |
| (Prick et al., 2024) | NR | The involved professional caregivers did not always keep track of which interventions received due to the changing duty schedules, staff movement, illness and work pressure among professional caregivers. | A volume check was also performed to ensure participants comfort with volume levels | 1. Music was delivered in a quiet, familiar and comfortable environment 2. Music selection according to patient’s personal preference 3. The professional caregiver monitored the intervention and regularly observed any reactions to the music | 1. Enable music program to be individualized 2. Music listening with headphones appears to be slightly more effective in reducing hyperactive behaviour. |
| (Grainger, 2024) | NR | NR | 1. Any participant removing the headphones was gently reminded to continue listening; 2. The retail cost for headphones were $9.99 on Amazon.com, total investment per participants was $50 for the MP3 player, headphones, and music | NR | NA |
| (Hillebrand et al., 2023) | Hearing impairment often occurs together with cognitive decline | NR | NR | 1. Individualized music listening is a low-cost and highly accepted intervention with few adverse effects | 1. Enable music program to be individualized; 2. BPSD were observed less frequently during the music listening |
| (Gulwani, 2022) | NR | High staff turnover rate, and incoming new staff's ability to participate in the project also affected the project. | 1. Residents with hearing loss will were excluded in the study 2. Activity staff and the DNP student monitored each participant session to replace headphones if removed. 3. The cost of this project for ten participants was less than $400 which included ten MP3 players, ten headphones, a storage box, and disinfectant. | 1. Before the implementation of music therapy, the DNP student provided a one-hour in-person educational session for nursing and another one-hour educational session for activity staff. 2. The music therapy is safe to implement, we observed no side effects, and it was easy to implement | 1. Enable music program to be individualized; 2. There was a significant decrease in agitation frequency and severity; 3. There was a significant decrease in aggression frequency and severity |
| (Gaviola et al., 2022) | 1. It can takes time for older adults to become accustomed to having something on their heads. 2. A small number of older adults reported discomfort using headphones, refusing to wear them or not wearing them for long periods of time, especially when they were agitated; 3. Although the headphones were padded, the pressure and size appeared to be a potential source of discomfort. | 1. Staff was frustrated about the equipment was not stored appropriately, or not turned off or not charged 2. Staff did not have the time to find the equipment or wait for it to be charged. | NR | 1. Staff members received the shortened training and they considered the training content is simple and did not have to be onerous; 2. The immediate positive responses of the participating older people with dementia to the music intervention inspired their family, guardian and staff members continued implementing the music intervention. | 1. Enable music program to be personalized 2. Older adults exposed to individualized music experienced a positive, calming effect that is mood enhancing, increases positive interactions with others, and evokes emotional responses and memories. |
| (Amor Gaviola et al., 2022) | 1. The cost of staff time on installing the headphones, checking the volume before listening, turn on the music and checking that the resident is in a comfortable position 2. The cost of staff time on removing, cleaning and storing the equipment after a music listening session | The cost of staff time on monitoring the resident's response during a music listening session, at least twice during a 30-minute session. | 1. The cost of headphones was AU$10.00 per item, which is relatively low. 2. Their music equipment can be reused. | 1. The family members and informal carers involved in the study received training before intervention; 2. Check that the resident is in a comfortable position; 3. The family members and informal carers delivered the music intervention to their relative during their visit 4. The music listening intervention via headphones was easy to implement and did not require a licence 5. The cost of the individualised music intervention described in this article – AU$222.56 per resident per year at 2022 values – is relatively low in comparison. | The cost of implementing the intervention was relatively low. |
| (McCreedy et al., 2021) | NA | NA | NA | NA | NA |
| (Lumsden, 2021) | Need sustainable funding to purchase more music devices and headphones | The educational training delivered virtually did not cover 100% staff due to the COVID-19 pandemic, which negatively impacted the engagement between researchers and staff | Low cost to buy headphones | 1. 68% staff completed the educational training and participated in a case study exercise presented during the training 2. Staff ongoing cooperation, commitment and dedication | 1. Enable music program to be individualized; 2. Individualized music delivered via headphones reduced the severity of dementia-related agitation |
| (Kwak et al., 2021) | 1. Music delivered via headphones program is very labour intensive. Lack of buy-in by direct care staff to initiate, deliver, and follow up with program. Without two interns it would have taken much longer. Delivering headphones and monitoring residents’ listening during delivery and at the end of each session also requires serious staff commitment. 2. Wearing headphones did not work for some residents (n = 86). Some residents simply did not like to wear headphones or earphones, and so speakers had to be used. 3. Headphones appeared to increase agitation or was overstimulating; 4. Headphones might not be suitable for residents with hearing aids. 5. Headphones lost or misplaced, which will lead to additional cost 6. Facilities will think they can just throw headphones on seniors to give them music, which could in turn devalue the work of a music therapist. | 1. Lack of or limited time for staff to implement and maintain the program; 2. Lack or inconsistency of volunteers; 3. Need to educate initial staff and new staff due to turnover; 4. Inadequate staffing; 5. Family was not supportive or helpful; 6. High staff turnover requires quite flexible and less burdening training to the staff; 7. Some residents complained that listening to music was too noisy and upset them; 8. Music equipment connected with headphones have problems with charging; 9. Music equipment connected with headphones like iPods was not user friendly for some seniors, which always need to ask for help. 10. Cost for buying music and iPod | 1. Financial support and donations to increase headphones. 2. Headphones were available in resident rooms, on the unit, open location for immediate access, easy to access 24/7; | 1. Staff have been provided training and support for the program; 2. Support of facility personnel (basically all staff including administrators), family, and volunteers to implement and maintain the program. 3. Support from volunteers | 1. Enable music program to be individualized; 2. Headphones can avoid distraction from noise; 3. Portability and use of headphones gave privacy without affecting others; 4. Caregivers can see participant's face when she has her headphones on and she looks genuinely happy, calm, and peaceful |
| (Kuot et al., 2021) | Some residents' occasional rejection of the equipment | 1. Resident’s musical taste and appreciation can change 2. Staff changes thus re-training of the new staff on the program implementation 3. High staff turnover requires quite flexible and less burdening training to the staff | The aged-care staff facilitated each resident listening to individualized digital music playlists using iPod device with a headphone | 1. Pre-recorded individualized music playlist is easy to access and can be conducted by nursing and direct care workers as part of their daily care regime. | 1. Enable the music program to be individualized; 2. Blocked noise and having the music playing through headphones made it more personal: The headphones make it specific to residents and they can't really hear what else is going on and can focus on the music they're listening to. 3. Created a calmer environment and reduced resistance to care among residents 4. Creating a less stressful environment for both residents and nursing staff. 5. Decreased agitation 6. Increased social interaction |
| (Harrison et al., 2021) | NR | Facility staff were tasked with multiple roles during the study and 3 LTC sites had to discontinue research because they lacked sufficient staff to cover the efforts required by participation. | 1. Residents who were able to tolerate headphones were allowed to participate 2. The staff were trained to implement the program headphones during the conduct of larger aspects of care 3. When music is heard, it can be shared through dance and vibration even during headphone use | 1. To participate in this study, the facility needed to have space for necessary equipment, stable personnel, and a willingness to participate in all aspects of the study 2. The long-term care staff were trained and supported by research staff in administering the intervention, housing equipment for the study in a safe, organized manner. | 1. Enable music program to be individualized; 2. Tended to decrease the agitation; 3. Older adults experienced a positive, calming effect that is mood enhancing, increases positive interactions with others, and evokes emotional responses and memories. |
| (Corrêa et al., 2020) | NR | NR | The headphone was used in the auditory pavilion, comfortable, in a volume of normal conversation (60-70 decibels) | 1. The music session took place in a suitable and silent room, previously prepared; 2. All the applicators were instructed to detect discomfort, such as signs of swearing, palpitation, negative features, crying sounds, negative speech by the elderly participant, among others. When any discomfort is noticed, the music could be replaced or the activity immediately stopped. | 1. Enable music program to be individualized; 2. Music delivered via headphones led to a higher percentage of expression of joy and surprise, 3. A greater number of trunk and head movements 4. A significant decrease in the severity of the symptoms of delirium after the interventions |
| (Weise et al., 2019) | 1. Two of 43 sessions discontinued the sessions due to repeatedly removing their headphones 2. Intensive involvement resulted in agitated | Battery of MP3-player dead | NR | 1. Prior to the start of the study, several meetings were hold to discuss the necessary adjustment of the planned procedure, inform the whole staff of the nursing home and relatives of nursing home residents about the study, which ensure the adverse responses to the music listening is minimized 2. Project staff or nursing home staff monitored the participants during the music intervention period and intervened when a negative reaction of a person with dementia was observed during the music listening or when a person with dementia expressed the wish to terminate listening in any way. | 1. Enable music program to be individualized; 2. participants had significantly better sleep quality |
| (Ihara et al., 2019) | NR | 1. Although each participant listened to personalized music using headphones, some individuals interacted with one another and/or the researchers while listening. | NR | 1. Participants were brought to another room to listen to their pre-programmed iPods. The room had comfortably seats 7-10 and maintained at a seasonally appropriate temperature to ensure the participants' physical comfort during the intervention; 2. The room door was closed, and only the researchers and participants were present in the room to minimize distraction. | 1. Enable music program to be individualized 2. Headphones enable participants to listen to their favourite songs repeatedly, or listened to a variety of songs. 3. Participants were able to retain the joy even after returning to normal activities. 4. The intervention helped participants appear more relaxed and calmer during the intervention 5. participants showed an increase in eye contact, eye movement, engagement and talkativeness, and a decrease in sleeping. |
| (Murphy et al., 2018) | 1. Ear buds were unusable as some participants wearing hearing aids; 2. Extra budget for broken or lost device replacement; 3. The music device was prominently displayed in the activities room which sought to prompt more routine utilization, while as needed usage has declined except for those residents who are able to ask for the device; 4. Residents required more attention from staff to keep headphones in place or to prevent falls from increased desire to stand and dance. | 1. Some residents did not respond favourably to music recommended by family members 2. Some residents who have been with the program for longer periods became less responsive to the same music; 3. Extra budget for new music purchases. 4. The iPod Shuffles and MP3 players are small and can be lost; 5. iPod Shuffles are no longer available, iPod Nanos have to be used to continue with the established iTunes library, but they are more expensive than the older Shuffles; 6. Administrative turnover at the facility required increased volunteer time spend at the facility and renegotiating program logistics. 7. Student volunteers spend trying songs and watching residents’ responses to identify the music preferences 8. Some resident initially refused music or showed withdrawn mood, often resulted from poor initial selection of music, and lack of personalization; 9. Maintaining the equipment was a challenge, including keeping the devices charged and stored properly: two devices were lost when they were in use and never properly returned to storage at the end of the day. | 1. Over-the-ear headphones were purchased for the participants as hearing aids made ear buds unusable; 2. An orientation was provided for ALF staff, with an emphasis on the MT’s role in passing out and collecting the devices; 3. Ongoing staff training and engagement to ensure proper use of the devices; 4. When music was played through an iHome speaker instead of headphones, the lack of personalized song selection led to unfavourable responses from residents and decreased responsiveness to the music recommended by family members; 5. Devices were placed in a designated location within a locked medication storage room. | 1. The facility administrator was contacted weeks before implementation to establish a trusting relationship that enabled program initiation; 2. The administrator prioritized enrolment of residents with the most severe cognitive impairment and behavioural symptoms, which addressed the facility’s greatest needs and helped ensure buy-in; 3. Several medical technicians, nursing assistants, and activities directors became program champions, which created ownership within the facility; 4. A 30 min service orientation was provided to staff, the simplicity of the program and the time spent in documenting the response were highlighted; 5. The facility advertises the program on its website. Each year 2–4 new medical student volunteers have been identified in the incoming class to adopt the program. Senior student volunteers’ mentor incoming volunteers regarding the importance of maintaining relationships with facility leadership and frontline staff champions, cultivating engagement through their visits, and providing in-service education. Incoming volunteers have latitude to continuously improve the program. | 1. Enable music program to be individualized; 2. Headphones create an immersive environment that minimized sensory distractions, enhancing the music's impact. 3. Headphone use increased the residents' socialization with staff and volunteers although this kind of interaction were not intentional (holding the arm of a resident once they decide to start walking or dancing with headphones on) 4. The iPods have been a huge help in calming residents during staff's busiest times 5. Staff endorsed feeling an ease in their work-flow and improved satisfaction with their work. |
| (Shiltz et al., 2016) | NR | NR | NR | 92 participants received hearing assessments by faculty members and undergraduate students with an audiology background | 1) Enable music program to be individualized  2) Delivered with headphones may reduce agitation. |
| (Kwak et al., 2016) | 1. It requires more staff time. The program relies on direct care staff to deliver the iPod/headphone as needed and based on a set schedule even during the day time. 2. Timing and frequency of use are difficult to track since the nursing staff are encouraged to put the headphones on the resident whenever they are awake and not participating in any activity and the staff rarely track the usage of headphones. The music therapist maintains equipment by doing weekly checks. 3. Wearing headphones did not work for some residents. Some residents simply did not like waring headphones or earphones, and instead speakers had to be used. 4. Some residents were unable to take headphones off when tired of listening. 5. Some residents showed more agitation or over stimulating due to using headphones. 6. Additional cost will be incurred to replace existing equipment, including headphones 7. Music therapist concern that facilities will think they can just throw headphones on seniors to give them music, which could in turn devalue the work of a music therapist, costing music therapists their jobs. | 1. A lack of buy-in from all levels of care staff and management. The program rely on direct care staff to deliver the iPod/headphone as needed and based on a set schedule even during the day time. 2. Additional cost incurred to replace existing equipment and buy new songs. 3. iPod have problems in charging 4. Residents often had trouble using iPods and dock stations independently they would get especially frustrated with volume control 5. Residents prefers quiet environment or not into music 6. Have difficulties in keeping track of all equipment and keeping the iPod charged 7. iPod is too small for residents to use. 8. iPod doesn’t have GPS which is difficult to locate and collect 9. Difficult to identify preferred songs for the playlist 10. Families were not supportive or helpful in music playlist development and selection | 1. Headphones were easy for all staff to use and store away; 2. Financial/donations enable staff always have iPod and headphones supply on hand 3. Accessibility of equipment (Available in resident rooms, on the unit, open location for immediate access, easy to access 24/7); | 1. Providing training and support for M&M 2. Facility personnel (all staff including administrators) positively involved 3. Family involvement (bringing music, donating shuffles to use, putting the iPod on, being supportive of the program) 4. Support from volunteers | 1. Enable music program to be individualized; 2. Headphones blocked noise 3. Enjoyment and improved mood with caveats that neither the music, nor using headphone or iPhone were observed in residents. 4. Reduced anxiety or stress during transitioning into the nursing home life 5. Provide comfort and relieve pain 6. Structured other individual or group activities for residents who are socially isolated |
| (Locke & Mudford, 2010) | Attenuation (i.e., blocking) of self-generated or environmental noise with inactive headphones was ineffective at reducing vocalizations. | NR | 1. The volume was set to the level considered by his care staff to be a comfortable and safe level 2. The headphones were preferred because participant was not required to sit down to listen to the music but allowed participant to walk and listen to music. 3. The headphones were preferred because some other residents complained about being disturbed in participant's ambient music condition | The intervention required less expertise and resources than the implementation of strictly behavioural procedures | 1. Music was played to Mr S via headphones, visitors and staff commented on how peaceful it was compared with their experiences before an effective intervention was discovered. 2. The intervention was effective only during the time when the music was playing, i.e., there were no carry-over effects. Music was the essential component instead of headphones. 3. Headphones at least allow participants to spend time in the lounge area in the company of others without being the target of verbal and physical abuse. |
| (Guetin et al., 2009) | NR | NR | Patients with hearing aids were not permitted to participate. | 1. The patients were either in a supine position or seated in a comfortable armchair. They were also offered a mask so as to avoid visual stimuli, thus encouraging them to concentrate on the music; 2. Patients were offered a mask so as to avoid visual stimuli, thus encouraging them to concentrate on the music; | 1. Enable music program to be individualized; 2. Reduce symptoms of anxiety and depression. |
| (Garland et al., 2007) | 1. Some participants became more agitated and removed the headphones indicating resistance to the intervention in certain cases. 2. There was some family members' reluctance to participant or refusal to wear headphones | NR | Residents were excluded from the study if residents’ refusal to wear headphones on three consecutive trials | 1. Generating personally directed sensory input presumably captured residents' interest more than the background commotion heard in many nursing homes. | 1. Enable music program to be individualized; 2. Alleviate agitation in nursing home residents with dementia to a modest and variable degree. |
| (Ragneskog et al., 2001) | The patient became irritated a few times during the intervention when the researcher adjusted his headphones, but the irritation only lasted for a few seconds | NR | NR | NR | 1. Headphones can avoid disturbing other patients; 2. Headphones can block noises 3. Patient's picking behaviour tended to diminish when he listened to the music |
